# Integrated tumor stromal features of hepatocellular carcinoma reveals two distinct subtypes with prognostic/predictive significance

**DOI:** 10.18632/aging.102064

**Published:** 2019-07-12

**Authors:** Wei Li, Jun Han, Kefei Yuan, Hong Wu

**Affiliations:** 1 Department of Liver Surgery and Liver Transplantation Center, West China Hospital, Sichuan University, Chengdu 610041, China; 2 Department of Critical Care Medicine, Sichuan Provincial Hospital for Women and Children, Chengdu 610045, Sichuan Province, China

**Keywords:** hepatocellular carcinoma, tumor stromal type, prognosis, lasso COX, nomogram

## Abstract

Current clinical classification of hepatocellular carcinoma (HCC) is unable to predict prognosis efficiently. Our aim is to classify HCC into clinically/biologically relevant subtypes according to stromal factors. We detected seven types of stromal features in tumors from 161 HCC patients by immunohistochemical staining and Hematoxylin-eosin staining. Five stromal features were selected out of seven types of stromal features to construct stromal type based on LASSO COX regression model. Then, integrating multiple clinicopathologic characteristics and stromal type, we built two nomograms for overall survival (OS) and disease-free survival (DFS). Further validation of the stromal type and nomograms were performed in the testing cohort (n = 160) and validation cohort (n = 120). Using the LASSO model, we classified HCC patients into stromal type A subgroup (CD34^low^TIL-stromal-ratio^high^Stromal-tumor-ratio^low^α-SMA^weak^Stroma^mature^) and stromal type B subgroup (CD34^high^TIL-stromal-ratio^low^Stromal-tumor-ratio^high^α-SMA^strong^Stroma^immature^). The stromal type was an independent prognostic factor for OS and DFS in the training, testing and validation cohorts. Two nomograms (for OS and DFS) that integrated the stromal type and clinicopathologic risk factors also showed good predictive accuracy and discriminatory power. In addition, immune cell recruitment in the tumor microenvironment (TME) was conditioned by the tumor stromal type. In conclusion, the newly developed tumor stromal type was an effective predictor of OS and DFS. Furthermore, the stromal type is associated with the immune phenotype in the TME.

## INTRODUCTION

Hepatocellular carcinoma (HCC) is the fifth most common cancer and the second most lethal cancer worldwide [1, 2]. Tumor staging systems are essential to classify patients in different risk groups based on the prognostic factors and to guide the therapeutic approaches. The American Joint Committee on Cancer staging system (TNM) and Barcelona Clinic Liver Cancer (BCLC) classification are used for routine prognostication and treatment allocation among patients with HCC, but neither provides substantial predictive value [3–5]. Currently, several biomarker-combined (e.g., α-fetoprotein) staging systems for HCC were established with good predictive ability [6, 7]. However, these staging systems still need to be validated in further studies.

The tumor microenvironment (TME) contains immune cells, tumor vasculatures and lymphatics, as well as fibroblasts and other extracellular matrix and soluble proteins such as cytokines and growth factors [8–10]. For many types of cancer including HCC, the composition of the TME is heterogeneous [8, 11]. For example, in breast tumors, some exhibit a poor tumor infiltration lymphocytes (TIL), while others are highly infiltrated by immune cells [12]. The long-term prognosis of tumors is determined by the genetic and epigenetic modifications of the transformed cells and also by the interactions of the malignant cells with their TME [10, 13]. The role of the non-malignant cells in the TME is complex with studies supporting both tumor-promoting and tumor-suppressing functions [13]. The rich TILs was found to be related to a better prognosis in many types of tumor [12, 14]. In addition, different amount, structure and activity of the fibrotic stroma was also found to be associated with the tumor progression and prognosis [12, 14–16]. A majority of patients developed HCC on a cirrhotic liver background and the increased extracellular matrix could significantly alter the TME and metabolism of HCC tumors. Finally, metabolic reprogramming of HCC cells could lead to the tumorigenicity and aggressiveness of HCC [17, 18]. Additionally, desmoplastic tumor stroma has been proposed to limit the entrance of drugs, influence tumor metastatic features, and alter the immune milieu relevant to immunotherapy [19]. Besides, the microangiogenesis and lymphangiogenesis could also play important roles in modulating the TME [9, 20].

Given the heterogeneity of the tumor stroma, it will be critical to understand the inter-relationship between stroma, neoplastic cells, and immune cells, which is significant for selection of immunotherapies (e.g., PDL and PDL1 blockade treatment) in patients with HCC. In addition, integrating stroma-related biomarkers into a model would substantially improve the prognostic power. In the present study, we aimed to characterize and stratify the tumor stoma of HCC according to the features of stromal components including TIL area, stromal volume, stromal maturity, stromal activity, stromal microvascular density (MVD) and lymphatic vessel density (LVD).

## RESULTS

### Construction of stromal score and definition of tumor stromal type

**Figure 1:**
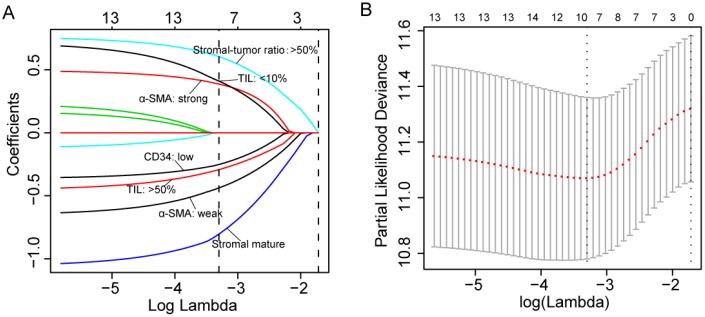
(**A**) LASSO coefficient profiles of the five selected stromal features. A dashed vertical line is drawn at the value (logγ=-3.3) chosen by 10-fold cross-validation. (**B**) Partial likelihood deviance for the LASSO coefficient profiles. A light dashed vertical line stands for the minimum partial likelihood deviance. A dashed vertical line stands for the partial likelihood deviance at the value (logγ=-3.3).

**Figure 2:**
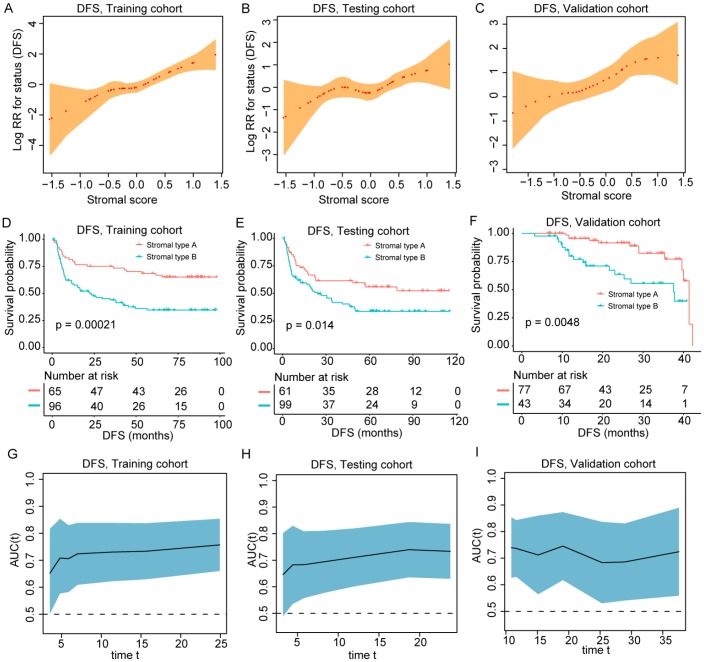
(**A**–**C**) The restricted cubic spline of the stromal score in training and validation cohorts (DFS). (**D**–**F**) Patients with stromal-type B had significantly worse disease-free survival than patients with stromal type A in training and validation cohorts. (**G**–**I**) The stromal score had acceptable predictive ability in all three cohorts. DFS, disease-free survival; RR, risk ratio; AUC, area under the receiver operating characteristic curve.

A total of 441 patients was enrolled in this study including 161 in the training cohort, 160 in the testing cohort and 120 in the validation cohort. Of the 441 patients included in this study, 368 (83.4%) were men, and the mean (SD) age of all patients was 50.7 (12.4) years. Other clinical features regarding etiology, tumor stage and liver function were shown in Table 1 and Supplementary Table 2. The representative images of the seven features were shown in Supplementary Figures 1–3. The optimal cut-off values (generated by X-tile plots) were used to divide CD31 (cut-off value: ≥5 vessels/mm^2^), CD34 (cut-off value: ≥17 vessels/mm^2^) and podoplanin (cut-off value: ≥2 vessels/mm^2^) into high and low expression group in the training cohort. Supplementary Table 3 showed the results of the univariate analysis between each of the seven features and the survival in the training cohort. We utilized a LASSO COX regression model to build a prognostic classifier (based on disease-free survival) in the training cohort, which integrated five features out of the seven parameters (Figure 1). Using the coefficients derived from the LASSO COX regression models, we then constructed a formula to calculate for each patient. This score is based on their personalized levels of the five features, where stromal score = (−0.437 × α-SMA: weak) + (0.387 × α-SMA: strong) − (0.804 × stromal maturity: mature) + (0.605 × stromal-tumor ratio: ≥ 50%) + (0.411 × TIL-Stromal ratio: < 10%) − (0.292 × TIL-Stromal ratio: ≥ 50%) − (0.252 × CD34: < 17 vessels/mm^2^). In this formula, the other expression status was equivalent to 0 (α-SMA: moderate, stromal maturity: immature and intermediate, stromal-tumor ratio: < 50%, TIL-Stromal ratio: 10%-50% and CD34≥ 17). Using X-tile plots, we classified patients into a stromal-type A and stromal-type B group with a stromal score of -0.03 as the cut-off value. Restrictive cubic spline functions of stromal scores in the training, testing and validation cohorts showed that the stromal score presented linear profiles (Figure 2A–2C and Supplementary Figure 4A–4C).

**Table 1:** Clinicopathologic characteristics of patients in the training and testing cohort.

**Variables**	**Training cohort (n =160)**	**Testing cohort (n = 161)**	**P**
Age, years	51.5 ± 12.0	49.7 ± 13.5	0.203
Gender			0.481
Male	134 (83.8%)	130 (80.7%)	
Female	26 (16.2%)	31 (19.3%)	
HBV infection			0.415
Negative	18 (11.2%)	23 (14.3%)	
Positive	142 (88.8%)	138 (85.7%)	
HBV-DNA, IU/mL			0.739
<10^3^	47 (39.5%)	45 (41.7%)	
≥10^3^	72 (60.5%)	63 (58.3%)	
AFP, ng/mL			0.539
<400	92 (57.5%)	98 (60.9%)	
≥400	68 (42.5%)	63 (39.1%)	
Preoperative ALT, IU/L	50.1 ± 40.8	45.8 ± 36.9	0.179
Preoperative AST, IU/L	49.4 ± 31.7	47.0 ± 31.2	0.168
ALBI Grade ½			0.660
Grade 1	110 (69.2%)	115 (71.4%)	
Grade 2	49 (30.8%)	46 (28.6%)	
FIB-4 score			0.167
Grade 1	29 (18.1%)	37 (23.0%)	
Grade 2	58 (36.2%)	67 (41.6%)	
Grade 3	73 (45.6%)	57 (35.4%)	
Tumor number			0.979
Single	130 (81.2%)	131 (81.4%)	
Multiple	30 (18.8%)	30 (18.6%)	
Tumor size, cm	6.0 ± 3.6	5.8 ± 3.3	0.497
AJCC-TNM Stage			0.825
Stage I	75 (46.9%)	81 (50.3%)	
Stage II	44 (27.5%)	41 (25.5%)	
Stage III	41 (25.6%)	39 (24.2%)	
BCLC Classification			0.887
A	125 (78.1%)	127 (78.9%)	
B	26 (16.2%)	27 (16.8%)	
C	9 (5.6%)	7 (4.3%)	
Tumor differentiation			0.617
Good	92 (57.5%)	97 (60.2%)	
Poor	68 (42.5%)	64 (39.8%)	
MVI			0.783
No	104 (65.0%)	107 (66.5%)	
Yes	56 (35.0%)	54 (33.5%)	

HBV, hepatitis B virus; AFP, alpha fetoprotein; ALT, alanine aminotransferase; AST, aspartate aminotransferase; ALBI, albumin-bilirubin; FIB-4, Fibrosis 4 Score; AJCC, American Joint Committee on Cancer; BCLC, Barcelona Clinic Liver Cancer; MVI, microvascular invasion.

### Association of tumor stromal type with patient survival

The median follow-up time of the current study was 35.1 months (range 1.0-114.6 months). As shown in Figure 2D–2F and Supplementary Figure 4D–4F, patients in stromal type B group had significantly worse OS and DFS than those in the stromal type A group in two cohorts. Univariate COX regression analysis identified tumor stromal type was a statistically significant factor associated with OS and DFS (Supplementary Tables 4–6) in the training, testing and validation cohorts. In the training cohort, the 1-, 3- and 5-year DFS rates for stromal type A and stromal type B were 79.7%, 73.4% and 68.5%; 58.8%, 43.9% and 34.7%, respectively. The 1-, 3- and 5-year OS rates were 95.4%, 89.0% and 74.1% for stromal-type A, and 79.3%, 52.0% and 40.2% for stromal-type B, respectively. In the present study, we calculated AUC to confirm the predictive accuracy of the stromal score. As shown in Figure 2G–2I and Supplementary Figure 4G–4I, the stromal score had acceptable predictive ability in all the training, testing and validation cohorts.

**Figure 3:**
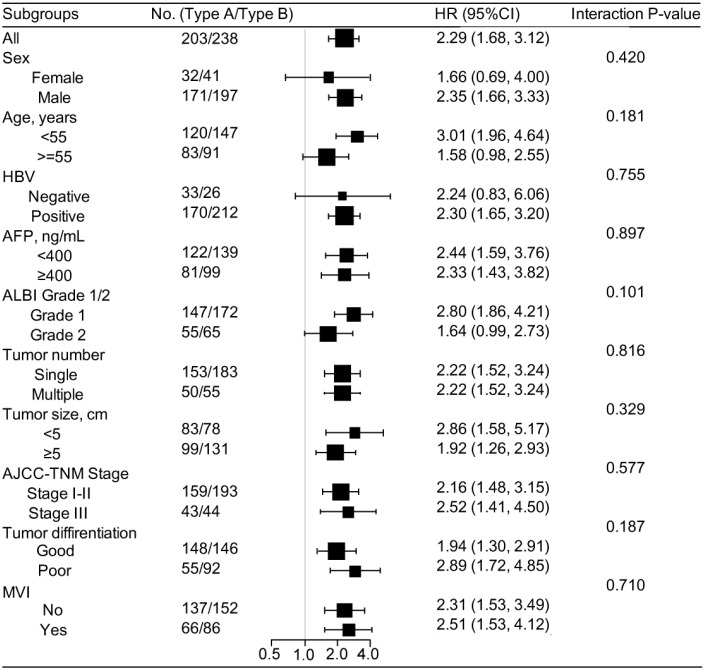
**Stratified analysis based on clinicopathologic features (disease-free survival).** In subgroup analyses, all identified confounding factors were adjusted except for the factor that the subgroup was based on. HBV, hepatitis b virus; AFP, alpha fetoprotein; ALBI, albumin-bilirubin; AJCC, American Joint Committee on Cancer; MVI, microvascular invasion.

The multivariable COX analyses demonstrated that stromal type was an independent prognostic factor for OS and DFS in all training, testing and validation cohorts (Table 2 and Supplementary Tables 7–8). To further evaluate the prognostic value of the stromal type, we compared OS and DFS between stromal type A and B with Kaplan-Meier survival analysis among different subgroups in all three cohorts (Supplementary Figures 5–6). In most of the subgroups, patients with stromal type A showed better OS and DFS than those with stromal type B. In addition, in multivariable analyses stratified by clinicopathologic features, we found that the salutary effects of stromal type A on OS (Supplementary Figure 7) and DFS (Figure 3) were consistent across all subgroups (all the interaction P values > 0.05).

**Table 2:** Multivariable analysis in the training cohort.

**Variables in the final model**	**Overall survival**			**Disease-free survival**		
	**HR**	**95%CI**	**P**	**HR**	**95%CI**	**P**
AFP, ≥400 ng/mL vs. < 400 ng/mL				1.43	0.90–2.25	0.126
ALBI Grade						
Grade 2 vs. Grade 1	1.63	1.03-2.58	0.039			
BCLC Classification						
BCLC-B vs. BCLC-A	1.18	0.65-2.15	0.592	1.67	0.96–2.90	0.070
BCLC-C vs. BCLC-A	3.06	1.17-8.02	0.023	3.05	1.33–7.01	0.008
MVI, Yes vs. no	1.71	1.07-2.73	0.025	1.55	0.96–2.53	0.075
Stromal type, type B vs. type A	2.88	1.75-4.76	<0.001	1.79	1.12–2.86	0.015

AFP, alpha fetoprotein; ALBI, albumin-bilirubin; BCLC, Barcelona Clinic Liver Cancer; MVI, microvascular invasion; HR, hazard ratio; CI, confidence interval.

### Construction and assessment of the nomograms

**Figure 4:**
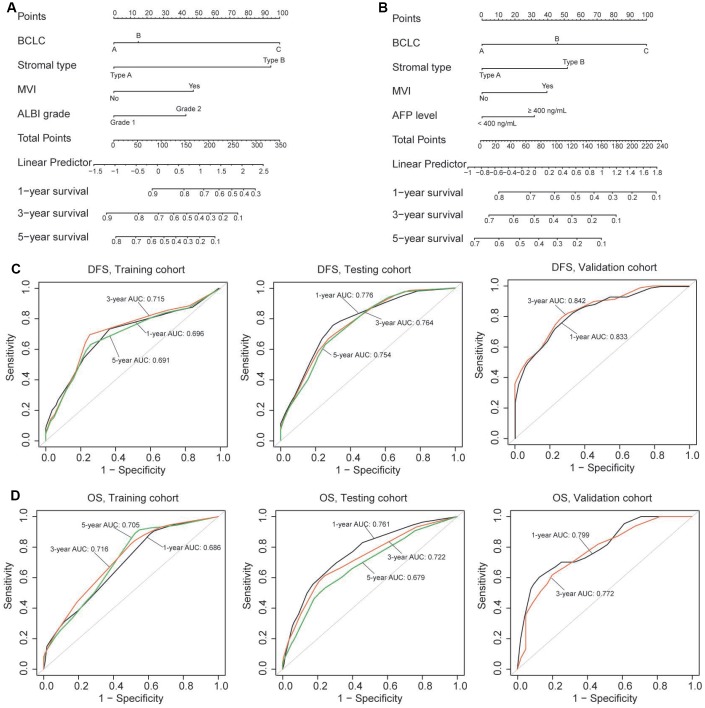
(**A**, **B**) Nomograms (for OS and DFS) that integrated the stromal type and clinicopathologic risk factors. To calculate the probability of status, sum up the points identified on the scale for all the variables and draw a vertical line from the total points scale to the probability scale. (**C**, **D**) ROC curves showing the predictive accuracy (1-, 3-, 5-year AUC) of the nomograms for OS and DFS in the three cohorts.

To provide a clinically relevant quantitative method to predict the probability of 1-, 3- and 5-year OS and DFS in patients with HCC, we constructed two nomograms (for OS and DFS) that integrated the stromal type and clinicopathologic risk factors (Figure 4A–4B; Table 2). The predictive accuracy (1-, 3-, 5-year AUC) of the nomograms in three cohorts is shown in Figure 4C–4D. The 1-, 3- and 5-year AUC for DFS was 0.696, 0.715 and 0.691, respectively, for the training cohort, and 0.776, 0.764 and 0.754, respectively, for the testing cohort. In the validation cohort, the 1- and 3-year AUC for DFS was 0.833 and 0.842, respectively. The 1-, 3- and 5-year AUC for OS was shown in Figure 4D. Calibration plots demonstrated that the nomograms performed well compared with the performance of an ideal model in all three cohorts (Supplementary Figure 8). When compared to the TNM (7^th^) and BCLC classification, our nomograms also showed better predictive accuracy (Supplementary Figure 9).

### Immune cell recruitment in the TME is conditioned by the tumor stromal type

**Figure 5:**
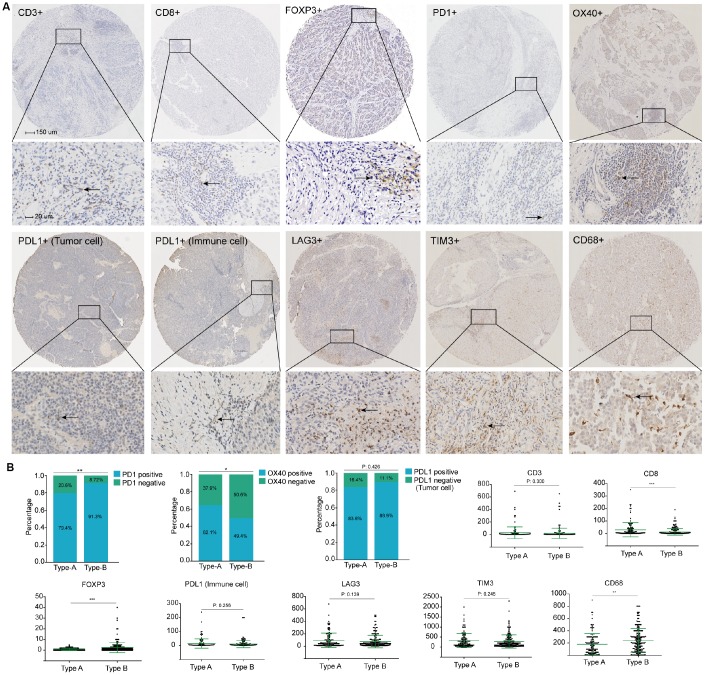
(**A**) Representative immunohistochemistry images of the ten immune markers. The bars (150 μm and 20 μm) were shown in the upper left figures. (**B**) Associations of stromal type with the immune markers. According to the data distributions, the optimal cut-off values for OX40 and PD1 were selected to perform comparison between groups, positive PD-L1 tumor cells staining was defined as more than 1% tumors cells staining on the membrane of the tumor cells. The others were compared by continuous data.

To evaluate how stromal biology may influence on the infiltrate and the levels of CD3+ T cells (representing total T cells) and CD8+ T cells, patients were stratified on the basis of stromal type. We found that stromal type A was associated with a higher number of infiltrating CD8+ T cells, whereas stromal type had no impact on the number of CD3+ T cells, suggesting that other subsets of lymphocytes account for the differences in TILs (Figure 5). Importantly, the number of macrophages (CD68) was strongly associated with stromal type, where stromal type A exhibited significantly decreased macrophage content (Figure 5).

Multiple immunosuppressive mechanisms are engaged in HCC. Distinct expression of the immune checkpoint molecules could be a reflection of differential immunosuppressive mechanisms in the TME. We assessed whether stromal type of the tumor was related to the immune checkpoint pathways. Finally, stromal type A was associated with higher number of PD1+ lymphocytes, while there was no correlation between stromal type and number of LAG3+ cells, TIM3+ cells, PDL1+ immune cells and PDL1+ tumor cells. Notably, stromal type A was related to a lower number of FOXP3+ T cells and OX40+ immune cells (Figure 5).

## DISCUSSION

Accurate prognostic evaluation is crucial for the selection of appropriate treatment for HCC. Integrating multiple biomarkers into a single model could improve the prognostic accuracy over that of a single model significantly [21]. HCC is clinically heterogeneous, with large variations in the clinical outcomes, even with the same TNM stage. Previous publications has introduced many molecular signatures to predict long-term survival in HCC patients [22–27]. These signatures, including genes, microRNAs, lncRNAs and epigenetic biomarkers, failed to be widely used clinically, as the variability of measurements in gene sequencings, inconsistencies in assay platforms, and the requirement for specialized analyses. Distinct from these studies focusing on molecular profiles of tumor cells, in the present study, a novel stromal type was developed comprising five selected stromal features of HCC, as a prognostic tool independent of other clinical features. Notably, our stromal classifier was established from evaluating stromal features thoroughly based on IHC and HE staining, which potentially can been widely applied in clinical practice [21, 40]. After adjustment for confounding factors, our stromal classifier was an independent prognostic factor for OS and DFS with good predictive accuracy in all three cohorts, even in different subgroups stratified by clinical variables (Figure 3 and Supplementary Figure 7). Consequently, the stromal classifier provides clinicians with a valid and reliable tool for better prediction of HCC prognosis. In addition, our stromal classifier consists of different parameters from TNM stage system, thus, patients classified with the same TNM stage might be able to be stratified into different risk groups according to the stromal classifier.

In contrast to other studies assessing tumor stroma of HCC [20, 28–30], our stromal type was established from evaluating stromal characteristics adequately based on the type of compositions in the stroma. Our classifier contained diverse stromal features (TIL-stromal ratio, stromal-tumor ratio, stromal maturity, stromal activation and stromal micro-vessels) and was constructed utilizing LASSO COX regression methods, which could significantly improve its predictive value [21]. In the ultimate formula, mature stroma, weak α-SMA, low microvascular density (CD34) and higher TIL-stromal ratio stood for favorable prognostic indicators while strong α-SMA, lower TIL-stromal ratio and higher stromal-tumor ratio stood for worse prognostic indicators. These observations were consistent with previous studies [14–16, 20, 30].

To improve the predictive accuracy of individual markers, we combined clinicopathologic characteristics with stromal classifier to predict patient survival. We divided HCC into stromal type A and stromal type B according to the cut-off value of the classifier (with large differences in long-term survival). Nomograms integrating information of stromal type, BCLC stage, MVI, ALBI grade and AFP level were established, and the nomograms had better prognostic values than TNM or BCLC stages alone in all three cohorts. Therefore, the nomograms provide clinicians with a more reliable instrument for better prediction of HCC prognosis.

Stromal type A consists of a CD34^low^TIL-stromal-ratio^high^Stromal-tumor-ratio^low^α-SMA^weak^Stroma^mature^ group. In this study, we found that stromal type A was correlated with higher number of CD8 T cells and lower number of macrophages. CD8+ cytotoxic T cells remain the mainstay of anti-tumor immunity, while the emerging literatures showed that the presence of tumor-associated macrophages were associated with more aggressive form of disease [8, 11, 21]. The different levels of these two types of immune cells indicated that stromal type A maintains a tumor-suppressing microenvironment in HCC. Interestingly, stromal type A was associated with lower level of FOXP3 and OX40 expression, which indicated that multiple immunosuppressive mechanisms were engaged in HCC. Both FOXP3 and OX40 are markers of regulatory T cells (Tregs), and they are crucial for the immune-suppressive function of Tregs [31]. Kinoshita et al., demonstrated that culture supernatant of cancer-associated fibroblasts from lung adenocarcinoma expressed higher levels of TGF-β and VEGF mRNA, and these cytokines can recruit Tregs to the TME [32]. Similarly, in this study, stromal type A had lower level of fibroblast activation (weak α-SMA) and a lower level of Tregs recruitment in the TME of the tumor. In addition, we observed a higher level of PD1 expression in stromal type A. However, due to the limited sample size, no associations were found between stromal type and PDL1 levels (both immune cell and tumor cell). These data illustrate that there is a profound diversity in the nature of immune response in HCC. Further studies are needed to illustrate whether the stromal type was associated with the responses of different types of immunotherapies.

Finally, we found that stromal type A was related to a higher level of E-cadherin expression and a lower level of vimentin expression (Supplementary Figure 10). This is consistent with the concept that tumor stromal type had a significant impact on the process of epithelial-mesenchymal transition (EMT) [13]. Patients with stromal type A had lower fibroblasts and higher level of immune cell infiltration in the stroma. Previous literatures demonstrated that tumor associated fibroblasts played a crucial role in constructing a metastatic niche and promoting tumor cell invasion and metastasis by secretion of chemokines and cytokines in the TME [33, 34]. Meanwhile, the “crosstalk” between stromal lymphocytes and EMT was also observed by the previous study [35, 36]. Chen et al. showed that the activation of EMT process was associated with the exhaustion of intratumoral CD8+ T lymphocytes [36]. Future studies should be carried out to illustrate the interacting traits between malignant cells and the TME, including immune cells, the vascular system and other stromal cells, which is necessary to develop multi-dimensional cancer therapies.

This study subjects to several limitations. First, all specimens were obtained from patients in the West China Hospital and there were no external validation cohort. Therefore, our results need to be validated in a prospective and larger cohorts. Second, the mechanisms behind the associations of stromal type with immune responses and tumor progression were not clearly elucidated in the present study, and further investigations may provide more information for better understanding of the roles of these stromal features in the development and progression of HCC. Third, the stromal features integrated in the classifier were incomplete (e.g., mesenchymal stem cells in the stroma was not included), thus, the stromal classifier may be further improved by including additional markers. Finally, we only used TIL-stroma ratio as a parameter of the classifier, while a detailed information of the infiltrated immune cells (e.g., B cells, dendritic cells and mast cells) was not included in the LASSO COX model.

In conclusion, this is the first study assessing the stromal features of HCC to predict the patient survival. We identified two stromal types by indicators of the tumor stroma. The stromal type could be a useful prognostic and predictive tool to identify HCC patients with better prognosis, thus, the stromal types might have significant implications for the postoperative personalized follow-up and treatment.

## PATIENTS AND METHODS

### Study population

The study was performed in accordance with the International Ethical Guidelines for Biomedical Research Involving Human Subjects (CIOMS) and conducted after approval by the ethic committee of the West China Hospital, Sichuan University. We obtained 441 HCC samples at the West China Hospital and patients were enrolled between June 2009 and December 2018. The data of patients who underwent hepatectomy for pathologically proven HCC at the liver surgery center were collected prospectively and analyzed retrospectively. We excluded patients if tumor samples or clinicopathological data were unavailable. Patients who received previous treatment including radiofrequency ablation and transcatheter arterial chemoembolization were also excluded. The resection procedure was performed as in our previous publication [37]. For the training and testing cohorts, data were obtained from 321 patients with HCC diagnosed between June 2009 and December 2014 at our hospital. Patients were randomly (by computer-generated random numbers) divided into training cohort (n = 161) and testing cohort (n = 160). In addition, we included an additional 120 patients in the internal validation cohort, with the same criteria as above, between January 2015 and December 2018.

Tumor staging was performed using the Barcelona Clinic Liver Cancer (BCLC) staging system and TNM staging system (7^th^). The Albumin-Bilirubin (ALBI) grade was utilized to evaluate liver function for patients with HCC [38]. FIB-4 score (a formula integrating age, platelet count, aspartate transaminase and alanine aminotransferase) was used to assess the cirrhosis of the background liver [39]. The median follow-up period was 56.4 months for the training group and 56.0 months for the validation group. Patients were followed up at a 2-month interval in the first one year after hepatectomy and at a 3-month interval thereafter. Overall survival (OS) was defined as the time from surgery to the last follow-up or death. The time of disease-free survival (DFS) was calculated from the date of surgery to the date of recurrence.

### Immunohistochemistry and Hematoxylineosin staining

The tissue microarrays were constructed by standard approaches with Formalin-fixed paraffin-embedded HCC tissues. Immunohistochemistry (IHC) and Hematoxylin-eosin (HE) staining were performed as described in Supplementary materials. On the basis of previous study findings [9, 11, 12, 15, 16, 20, 40], we selected seven prognostic stromal biomarkers to delineate tumor stroma including stromal-tumor ratio, TIL-stromal ratio, stromal maturity, stromal activity (α-SMA), CD31, CD34 and podoplanin expressions. Stromal-tumor ratio were divided into stroma-poor (proportion of stroma ≤50%) and stroma-rich (proportion of stroma >50%) groups on HE-stained TMEs (Supplementary Figure 1). The average TIL area was calculated according to a standardized evaluation tutorial. Briefly, the TIL-stromal ratio was calculated by area occupied by immune cells over total intratumoral stromal area (not the number of stromal cells) (Supplementary Figure 2). Large areas of central necrosis or fibrosis are not included in the evaluation. In addition, we only include mononuclear infiltrate (lymphocytes & plasma cells) and we do not include granulocytic infiltrate in areas of tumor necrosis. Patients were classified into three groups based on TIL-stromal ratio (group A: 0–10% stromal TILs; group B: 10%-50% stromal TILs; group C: 50%-90% stromal TILs) [41]. The stromal maturity was defined according to previous literature [16]. Fibrotic tumor stroma was classified as mature when composed of mature collagen fibres (fine and elongated fibres with fibrocytes stratified into multiple layers). Stroma was classified as immature fibrotic stroma when the collagen bundles was keloid-like, randomly orientated, and surrounded by myxoid stroma. The intermediate group was confirmed when the bands of collagen was randomly orientated, while with mature collagen fibres simultaneously (Supplementary Figure 1) [42]. In addition, CD31 and CD34 (microvascular marker), podoplanin (lymphatic vessel marker) and α-SMA (fibroblasts marker, represents stroma activation) were evaluated on the IHC-stained TMEs (Supplementary Figures 1 and 3).

Besides, The TMEs were stained with the following antibodies: CD3, CD8, CD68, FOXP3, PD-1, PD-L1, TIM-3, LAG3, OX40, E-cadherin, and vimentin. The detailed information of the primary antibodies for IHC is shown in Supplementary Table 1.

### Quantitation of the stained cells

The IHC outcomes were evaluated by two independent pathologists who were blinded to the clinical outcome. For immune cells and micro-vessels, to evaluate the density of stained cells, three respective areas of stroma were evaluated at × 200 magnification and the mean value was adopted. The MVD in tumor tissues of HCC was assessed by staining for CD31 and CD34. The LVD was evaluated by staining podoplanin. Any discrete cluster or single cell stained for CD31, CD34 and podoplanin was counted as one micro-vessel or lymph vessel (vessels/mm^2^). For immune cell (CD3, CD8, CD68, FOXP3, PD-1, immune cell PD-L1, TIM-3, LAG3 and OX40), E-cadherin and vimentin staining, the counts of all positive cells by immunostaining were changed into density as cells/mm^2^. Specially, positive PD-L1 tumor cells staining was defined as more than 1% tumors cells staining on the membrane of the tumor cells.

### Statistical analysis

All statistical tests were two-sided, and P < 0.05 was considered to be statistically significant. Continuous variables were presented as mean ± SD and tested by t-test or Kruskal-Wallis test. Categorical variables were expressed as number (%) and tested by Chi-square test or Fisher’s exact test. The survival curves were determined by the Kaplan-Meier method and compared by the log-rank test.

We utilized the LASSO COX regression model (10-fold cross-validation; logγ = −3.3) to select the most useful prognostic parameters out of all the HCC-associated stromal features, and then established a classifier based on multi-stromal features for predicting survival in the training cohort. The “glmnet” package was used to perform the LASSO COX regression analysis.

Multivariable COX proportional hazards regression models were employed to calculate Hazard ratios (HR) and 95% confidence intervals (CI) for comparisons between groups (stromal type A and type B). Confounders were selected based on the following commonly used criteria: the factor was associated with the main predictor (stromal features) or the dependent factor (patient survival), and it was not in the causal pathway between the outcomes and the main predictor. In adjusted models, we adjusted for covariates that changed HR or β by at least 10%, when they were added to or removed from the model [43]. And we also adjusted covariates with P values less than 0.2 in univariate analyses. In addition, clinically clear prognostic factors were also adjusted even they did not meet the above criteria. The variables that were included in the COX regression analysis were age, sex, ALT and AST level, HBV infection, HBV-DNA level, AFP level, ALBI grade, TNM stage, tumor differentiation, MVI and tumor stromal type. The interaction test was performed to detect the influence of each stratified factor on the relationship between stromal type and patient survival. P value for interaction less than 0.05 means interaction exists between that factor and the relationship.

Two nomograms were built up on the basis of the results of the multivariable COX proportional hazards regression models. A final model selection was performed by a backward step-down selection process with the Akaike information criterion. Based on the identified prognostic variables, the nomograms were established for predicting 1-, 3-, and 5-year OS and DFS after hepatectomy. The discriminatory capabilities of the nomograms were assessed with the receiver operating characteristic curve and the area under the receiver operating characteristic curve (AUC). Calibration of the model was evaluated by Harrell’s C statistics and the correlation coefficient between predicted and observed probabilities of death or tumor recurrence. All statistical analyses were performed by R (http://www.R-project.org) and EmpowerStats software (www.empowerstats.com, X&Y solutions, Inc. Boston MA).

## Supplementary Material

Supplementary Methods

Supplementary Figures

Supplementary Table
