# Supplementary material for: Integrated tumor stromal features of hepatocellular carcinoma reveals two distinct subtypes with prognostic/predictive significance
Source: Aging (Albany NY). 2019 Jul 12;11(13):4478–509. doi: 10.18632/aging.102064 (PMC6660041; doi:10.18632/aging.102064)
Supplement: Supplementary Methods [file aging-11-102064-s001.pdf]

SUPPLEMENTARY METHODS

Immunohistochemistry

Immunohistochemistry was performed according to standard protocols. Sections were baked at 60 ℃ for 40 minutes, de-waxed in xylene, and rehydrated in decreasing concentrations of ethanol. Prior to staining, the sections were subjected to endogenous peroxidase blocking in 3% of H_2O_2 solution in methanol for 15 min. Antigen retrieval was carried out by heating in a microwave for 15 min in citrate antigen retrieval solution (PH: 6). After incubated with monoclonal antibodies against CD3, CD8, CD68, FOXP3, PD-1, PD-L1, TIM-3, LAG3, OX40, E-cadherin, and vimentin overnight at 4°C and then incubated with a labeled polymer/HRP amplification system (ZLI-9018 and PV-6000, ZSGB-BIO, China) for 30 min. The immunoreaction was detected after treatment with diaminobenzidine chromogen for 1 minutes. All staining runs included a no-primary-antibody control. The antibody dilutions and antigen retrieval are shown in Supplementary Table 1. Immunoreaction images were viewed and captured by the Image-Pro-Plus 6.0 software.
