# Supplementary material for: Integrated tumor stromal features of hepatocellular carcinoma reveals two distinct subtypes with prognostic/predictive significance
Source: Aging (Albany NY). 2019 Jul 12;11(13):4478–509. doi: 10.18632/aging.102064 (PMC6660041; doi:10.18632/aging.102064)
Supplement: Supplementary Figures [file aging-11-102064-s003.pdf]

SUPPLEMENTARY FIGURES

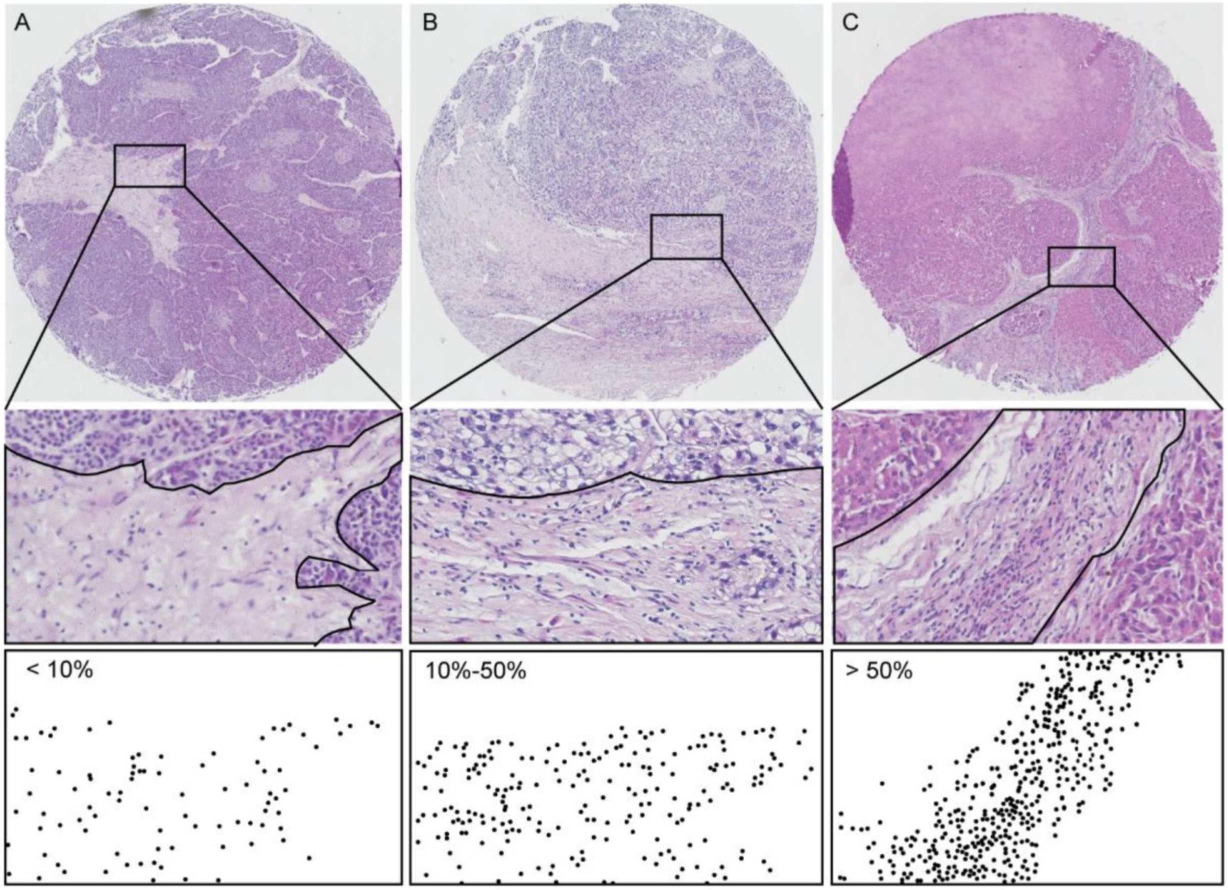

Supplementary Figure 2. With HE staining slices, the TIL-stromal ratio was calculated by area occupied by immune cells over total intratumoral stromal area (not the number of stromal cells). Patients were classified into three groups based on TIL-stromal ratio (group A: 0–10% stromal TILs; group B: 10%–50% stromal TILs; group C: 50%–90% stromal TILs).

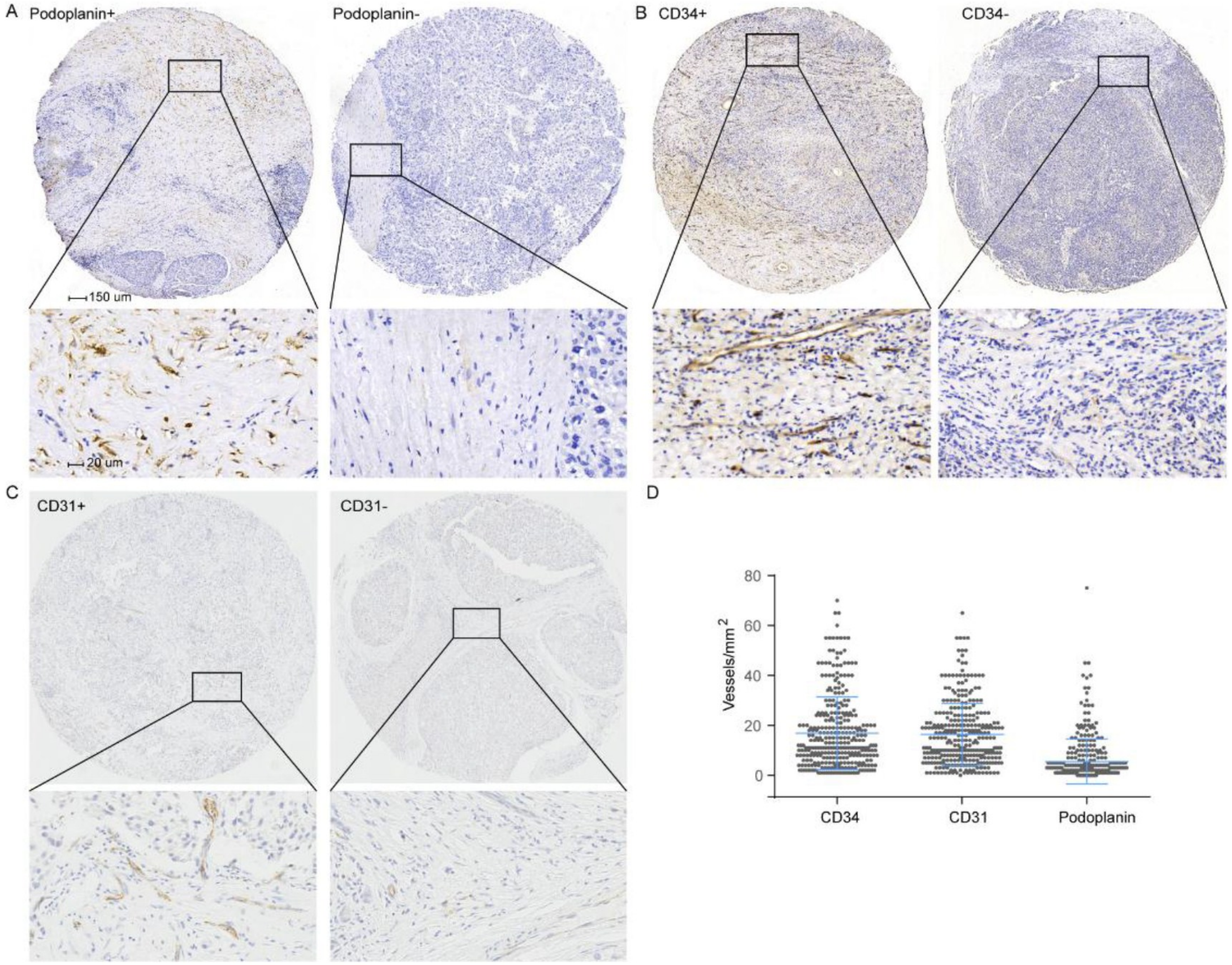

Supplementary Figure 3. (**A**–**C**) CD31 and CD34 (microvascular marker) and podoplanin (lymphatic vessel marker) were evaluated on the IHC-stained TMEs. D: The expressions of these three markers (vessels/mm^2).

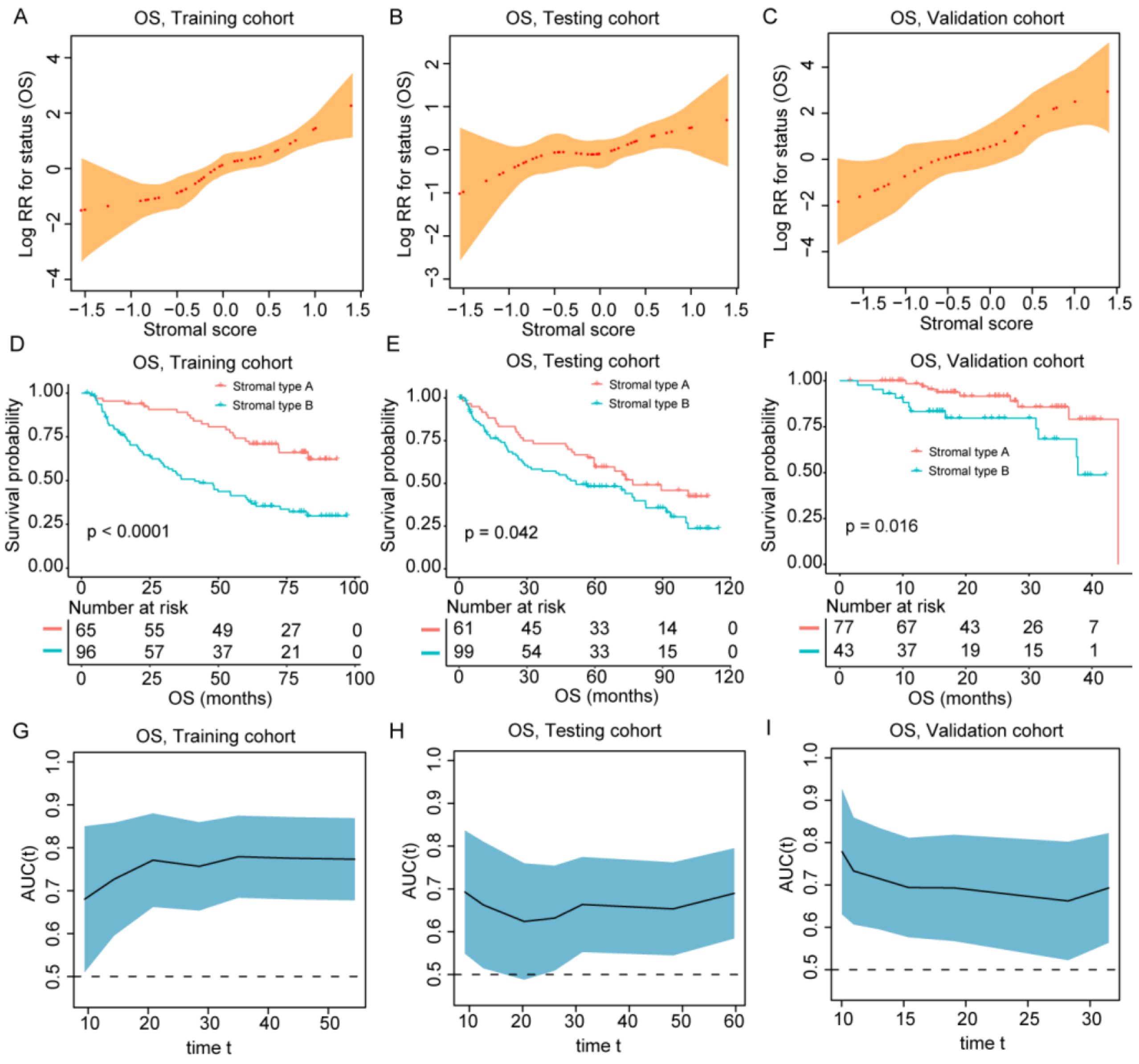

Supplementary Figure 4. (**A**–**C**) The restricted cubic spine of the stromal score in training and validation cohorts (OS). (**D**–**F**) Patients with stromal-type B had significantly worse overall survival than patients with stromal type A in training and validation cohorts. (**G**–**I**) The stromal score had acceptable predictive ability in the training, testing and validation cohorts. OS, overall survival; AUC, area under the receiver operating characteristic curve.

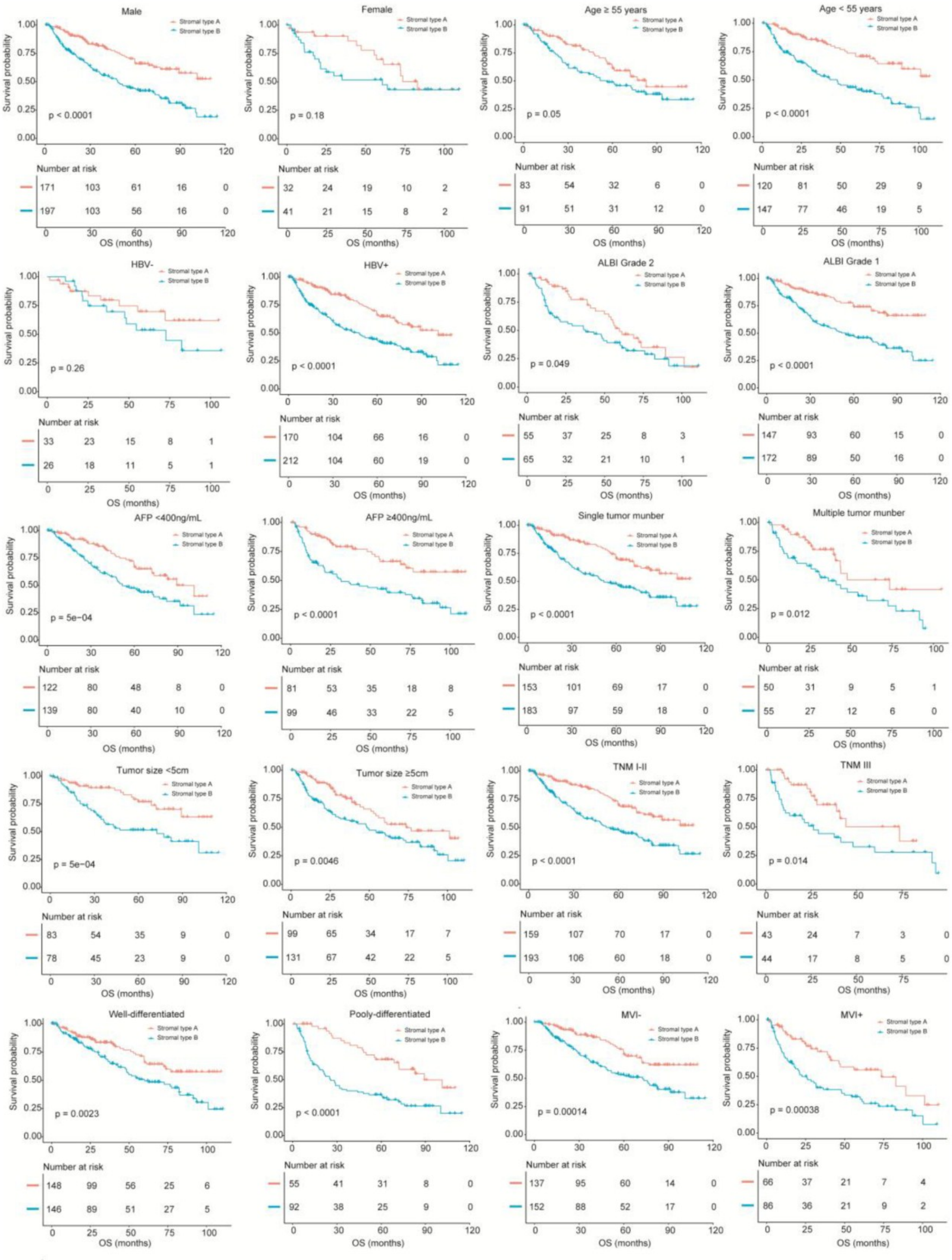

Supplementary Figure 5. Comparisons of overall survival between stromal-type subgroups with Kaplan-Meier survival analysis among different subgroups in the total cohorts.

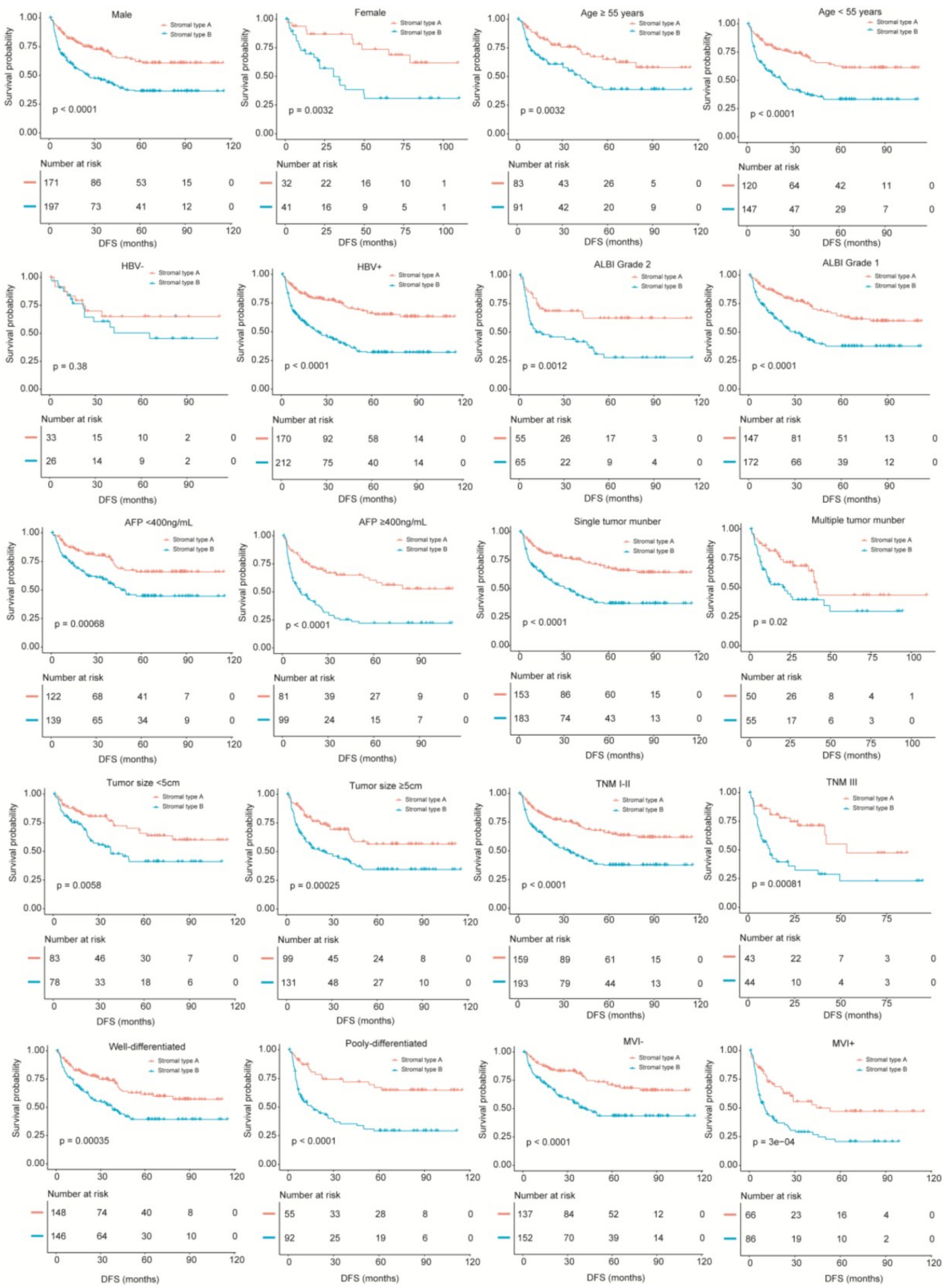

Supplementary Figure 6. Comparisons of disease-free survival between stromal-type subgroups with Kaplan-Meier survival analysis among different subgroups in the total cohorts.

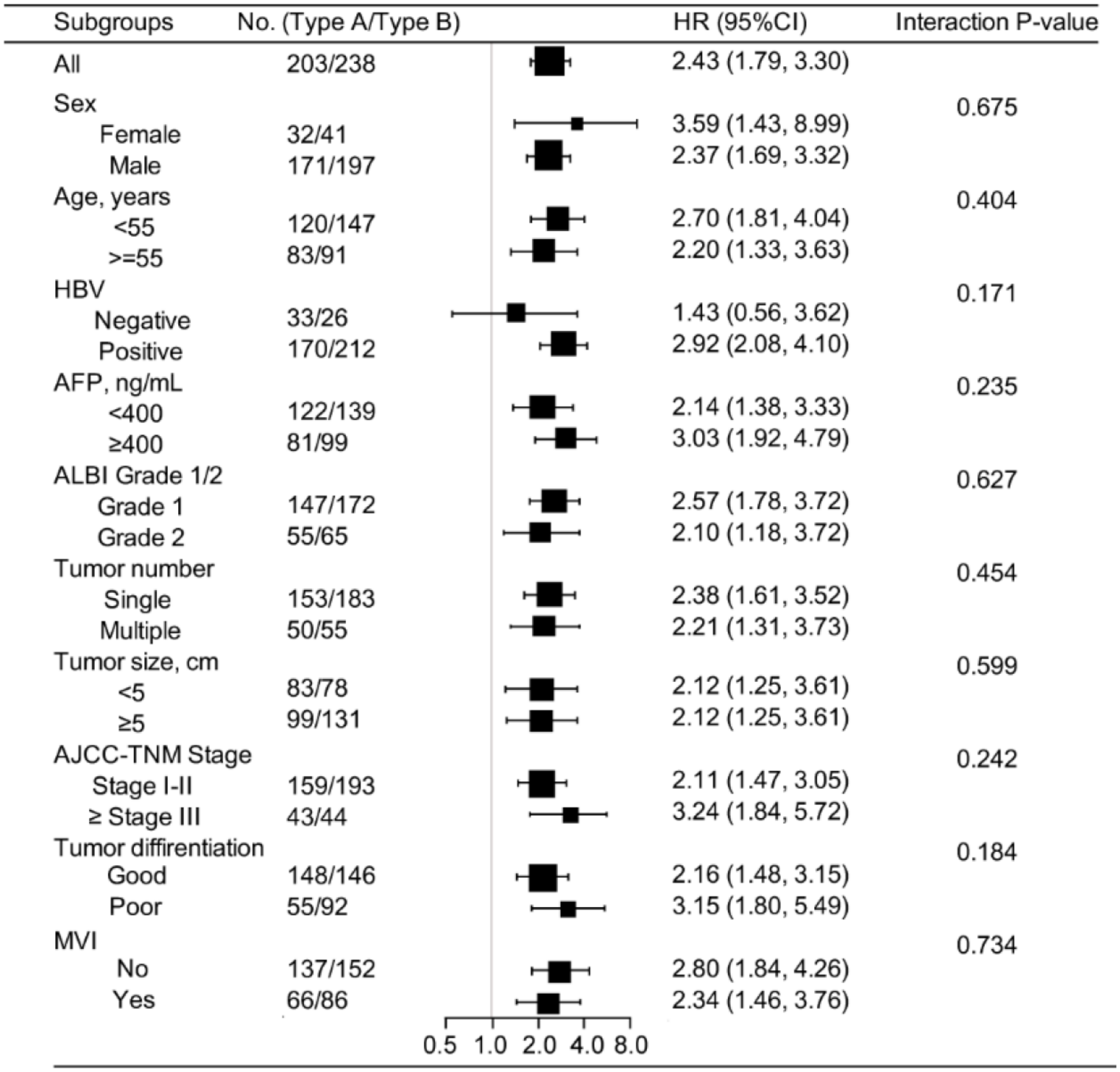

Supplementary Figure 7. Stratified analysis based on clinicopathologic features (overall survival). In subgroup analyses, all identified confounding factors were adjusted except for the factor that the subgroup was based on. HBV, hepatitis b virus; AFP, alpha fetoprotein; ALBI, albumin-bilirubin; AJCC, American Joint Committee on Cancer; MVI, microvascular invasion.

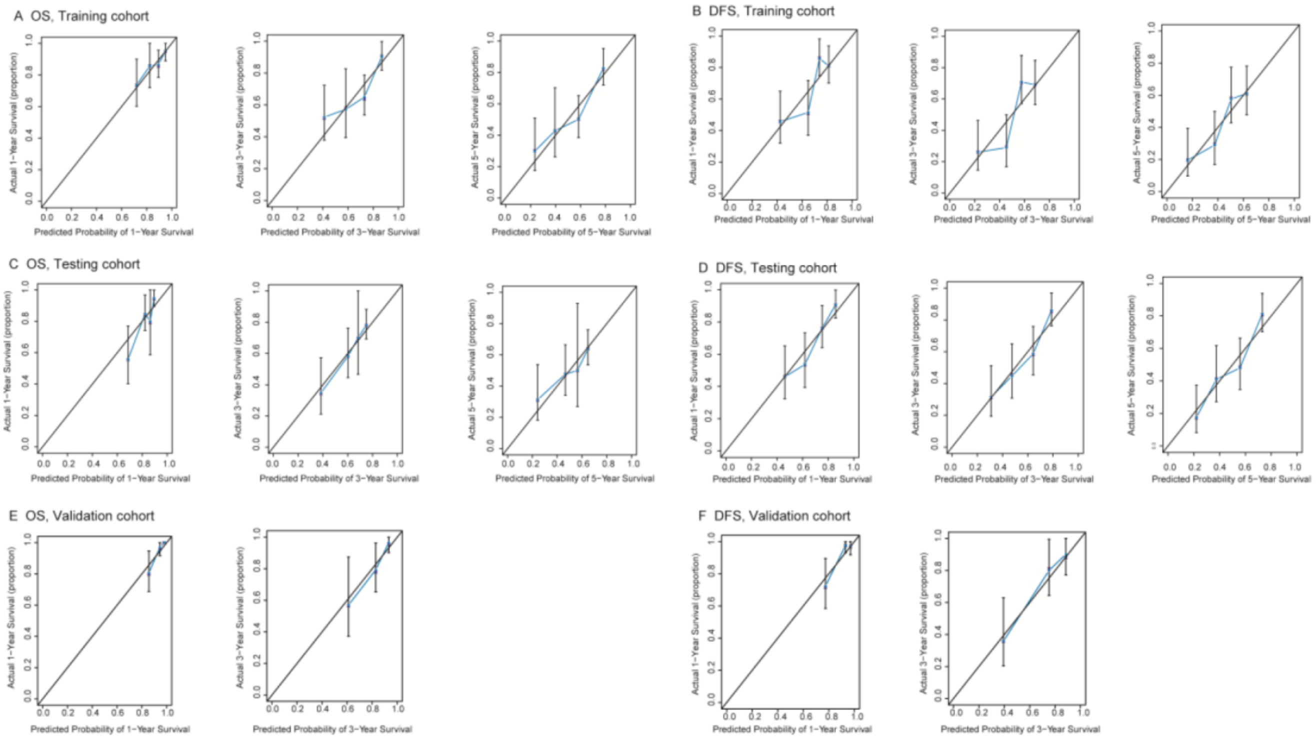

Supplementary Figure 8. Calibration plots demonstrated that the nomograms performed well for predicting both overall and disease-free survival compared with the performance of an ideal model in three cohorts.

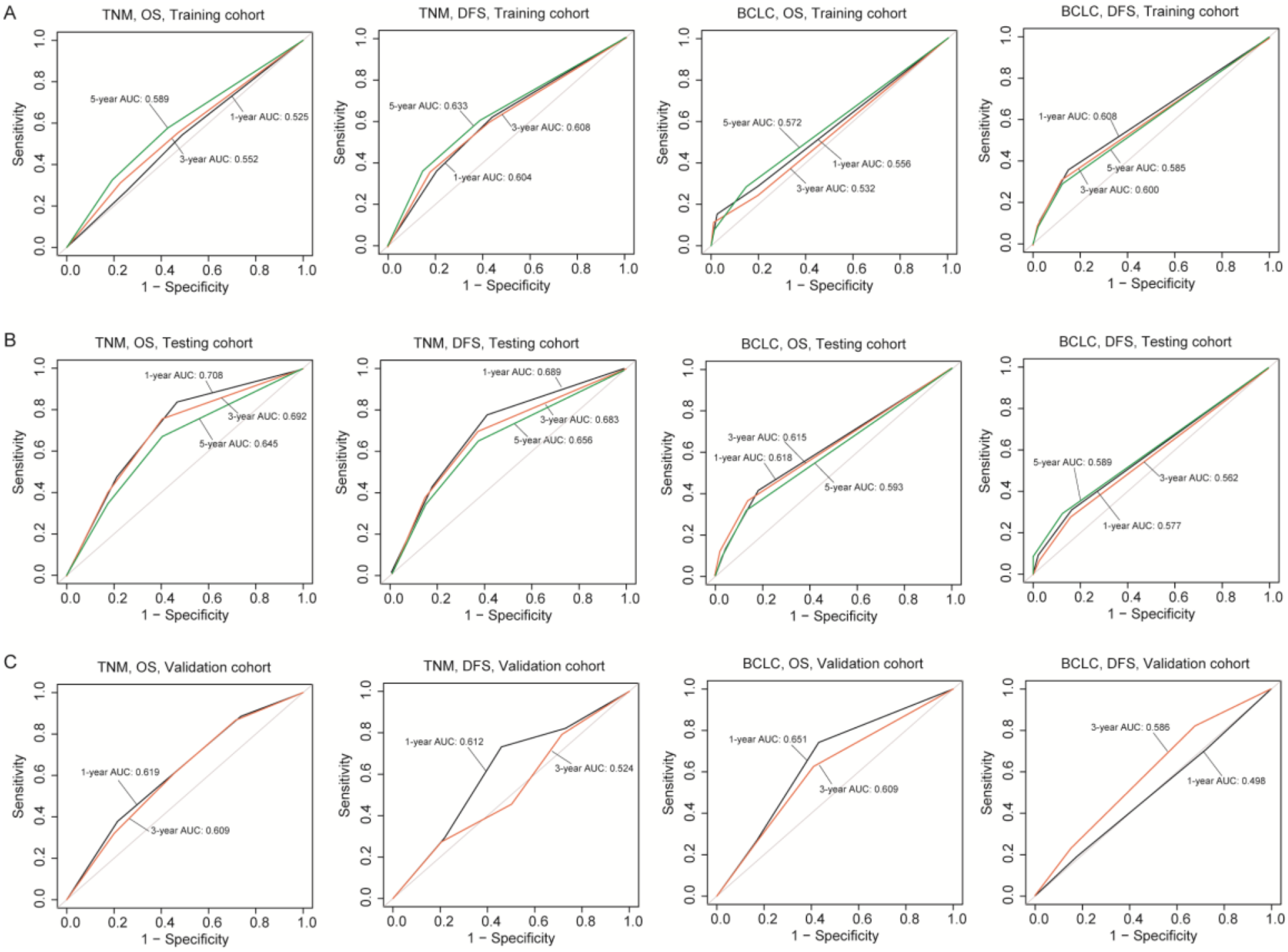

Supplementary Figure 9. ROC curves showing the predictive value (1-, 3-, 5-year AUC) of the TNM (7th) and BCLC classifications in the training, testing and validation cohorts.

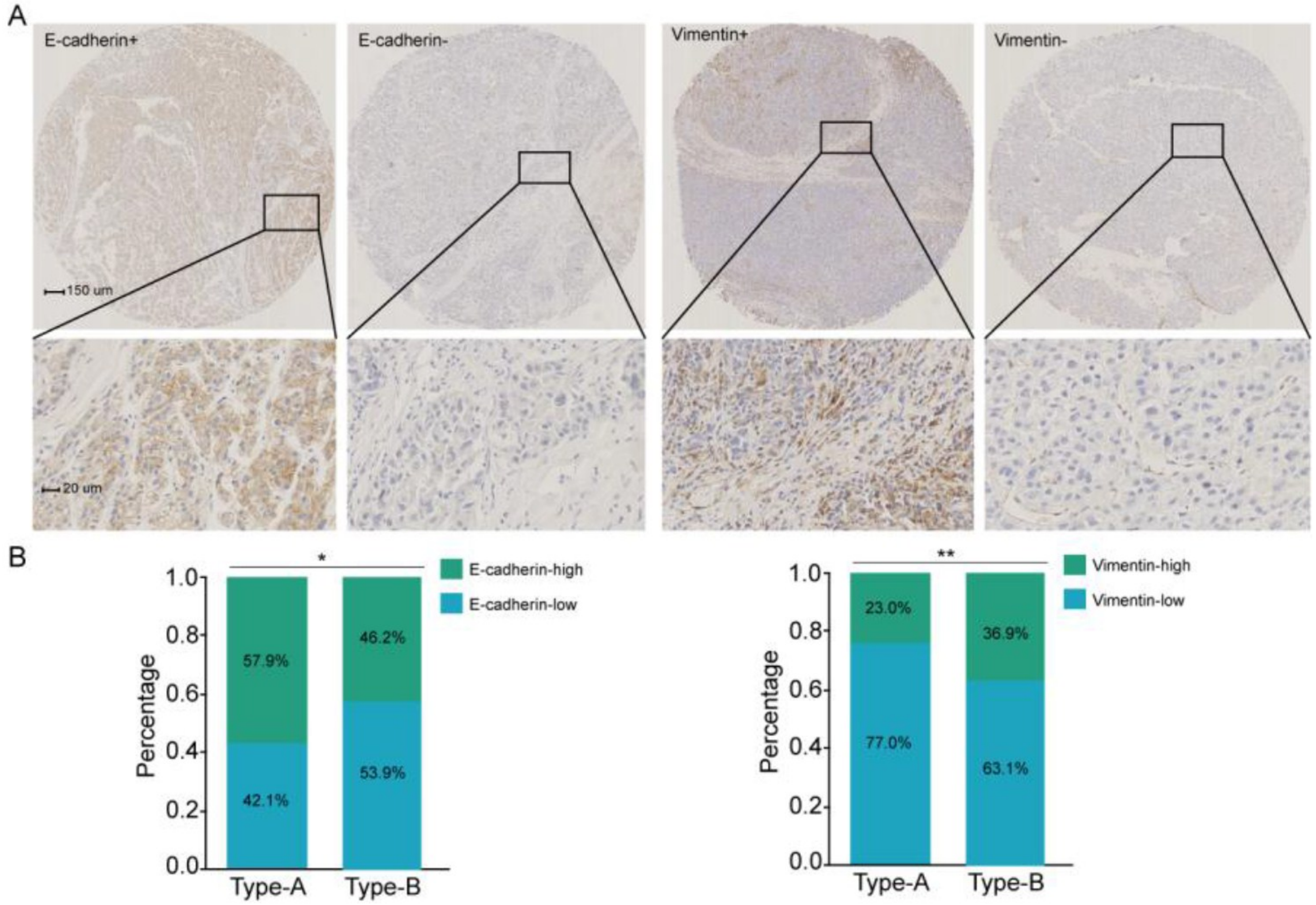

Supplementary Figure 10. (**A**) Representative immunohistochemistry images of E-cadherin and vimentin. (**B**) Stromal type A was related to a higher level of E-cadherin expression and a lower level of vimentin expression. The optimal cut-off values for E-cadherin and vimentin were selected to perform comparison between groups.
