# Supplementary material for: Integrated tumor stromal features of hepatocellular carcinoma reveals two distinct subtypes with prognostic/predictive significance
Source: Aging (Albany NY). 2019 Jul 12;11(13):4478–509. doi: 10.18632/aging.102064 (PMC6660041; doi:10.18632/aging.102064)
Supplement: Supplementary Table [file aging-11-102064-s002.pdf]

SUPPLEMENTARY TABLES

Supplementary Table 1. Antibody sources and staining conditions.

Markers	Antibody source	Species	Dilution	Antigen retrieval buffer
CD31	Abcam, ab28364	Rabbit monoclonal	1:200	Citrate buffer (pH 6.0) microwave 16min
CD34	Abcam, ab81289	Rabbit monoclonal	1:200	Citrate buffer (pH 6.0) microwave 16min
Podoplanin	Abcam, ab77854	Mouse monoclonal	1:200	Citrate buffer (pH 6.0) microwave 16min
α-SMA	Abcam, ab5694	Rabbit monoclonal	1:200	Citrate buffer (pH 6.0) microwave 16min
CD68	DAKO, M087601-2	Mouse monoclonal	1:200	Citrate buffer (pH 6.0) microwave 16min
FOXP3	Abcam, ab20034	Mouse monoclonal	1:100	Citrate buffer (pH 6.0) microwave 16min
CD3	Abcam, ab16669	Rabbit monoclonal	1:200	Citrate buffer (pH 6.0) microwave 16min
CD8	Abcam, ab33786	Mouse monoclonal	1:200	Citrate buffer (pH 6.0) microwave 16min
TIM3	R&D Systems, AF2365-SP	Goat monoclonal	1:100	Citrate buffer (pH 6.0) microwave 16min
LAG3	LifeSpan Bioscience, LS-B2237	Mouse monoclonal	1:100	Citrate buffer (pH 6.0) microwave 16min
PDL1	CST, #13684	Rabbit monoclonal	1:200	Citrate buffer (pH 6.0) microwave 16min
PDCD1	Abcam, ab52587	Mouse monoclonal	1:50	Citrate buffer (pH 6.0) microwave 16min
OX40	Abcam, ab119904	Rabbit monoclonal	1:100	Citrate buffer (pH 6.0) microwave 16min
E-cadherin	Santa Cruz, sc-8426	Mouse monoclonal	1:100	Citrate buffer (pH 6.0) microwave 16min
Vimentin	Santa Cruz, sc-6260	Mouse monoclonal	1:100	Citrate buffer (pH 6.0) microwave 16min

Supplementary Table 2. Clinicopathological characteristics of patients in the Validation cohort.

Variables	Validation cohort (n = 120)
Age, years	51.2 ± 11.1
Gender	
Male	104 (86.7%)
Female	16 (13.3%)
HBV infection	
Negative	18 (15.0%)
Positive	102 (85.0%)
HBV-DNA, IU/mL	
$<10^3$	45 (37.5%)
$\geq 10^3$	75 (62.5%)
AFP, ng/mL	
<400	71 (59.2%)
≥400	49 (40.8%)
Preoperative ALT, IU/L	41.7 ± 35.5
Preoperative AST, IU/L	45.6 ± 28.9
ALBI Grade 1/2	
Grade 1	94 (79.0%)
Grade 2	25 (21.0%)
FIB-4 score	
Grade 1	23 (19.2%)
Grade 2	57 (47.5%)
Grade 3	40 (33.3%)
Tumor number	
Single	75 (62.5%)
Multiple	45 (37.5%)
Tumor size, cm	
<5	43 (35.8%)
≥5	77 (64.2%)
AJCC-TNM Stage	
Stage I	47 (39.2%)
Stage II	31 (25.8%)
Stage III	42 (35.0%)
BCLC Classification	
A	66 (55.0%)
B	33 (27.5%)
C	21 (17.5%)
Tumor differentiation	
Good	105 (87.5%)
Poor	15 (12.5%)
MVI	
No	78 (65.0%)
Yes	42 (35.0%)

HBV, hepatitis B virus; AFP, alpha fetoprotein; ALT, alanine aminotransferase; AST, aspartate aminotransferase; ALBI, albumin-bilirubin; FIB-4, Fibrosis 4 Score; AJCC, American Joint Committee on Cancer; BCLC, Barcelona Clinic Liver Cancer; MVI, microvascular invasion.

Supplementary Table 3. Univariate analysis of the stromal features in three cohorts (disease-free survival).

Variables	Training cohort (n=161)			Testing cohort (n=160)			Validation cohort (n=120)		
	HR	95%CI	P	HR	95%CI	P	HR	95%CI	P
α-SMA									
Weak	1	1	1	1	1	1	1	1	1
Moderate	1.19	0.74–1.90	0.472	1.61	1.01–2.56	0.047	1.21	0.56–2.59	0.631
Strong	1.47	0.74–2.93	0.274	3.46	1.65–7.2	0.001	1.59	0.46–5.48	0.463
Stromal maturity									
Immature	1	1	1	1	1	1	1	1	1
Intermediate	0.99	0.62–1.58	0.972	1.16	0.72–1.85	0.546	0.41	0.12–1.43	0.161
Mature	0.39	0.19–0.81	0.012	0.41	0.18–0.91	0.028	0.25	0.06–0.96	0.044
Stromal-tumor ratio									
> 50%	1	1	1	1	1	1	1	1	1
≤ 50%	0.86	0.54–1.38	0.536	0.48	0.31–0.75	0.001	0.34	0.15–0.74	0.006
TIL-stromal ratio									
< 10%	1	1	1	1	1	1	1	1	1
10%–50%	0.81	0.51–1.28	0.364	0.79	0.49–1.27	0.337	0.23	0.08–0.65	0.006
> 50%	0.42	0.19–0.94	0.035	0.48	0.24–0.97	0.040	0.34	0.12–0.97	0.044
CD31									
Low (<5 vessels/mm^2)	1	1	1	1	1	1	1	1	1
High (≥5 vessels/mm^2)	1.12	0.62–2.02	0.711	1.45	0.67–3.16	0.345	0.95	0.33–2.75	0.929
CD34									
Low (<17 vessels/mm^2)	1	1	1	1	1	1	1	1	1
High (≥17 vessels/mm^2)	1.55	0.98–2.46	0.063	1.53	0.99–2.39	0.058	1.84	0.89–3.79	0.099
Podoplanin									
Low (<2 vessels/mm^2)	1	1	1	1	1	1	1	1	1
High (≥2 vessels/mm^2)	0.94	0.61–1.46	0.792	0.94	0.60–1.45	0.769	0.56	0.27–1.13	0.103

HR, hazard ratio; CI, confidence interval.

Supplementary Table 4. Univariate analysis in the training cohort.

Variables	Overall survival			Disease-free survival		
	**HR**	**95%CI**	**P**	**HR**	**95%CI**	**P**
Age, years	1.00	0.98–1.02	0.917	1.00	0.98–1.02	0.914
Gender, female vs. male	0.90	0.52–1.55	0.704	1.35	0.78–2.33	0.282
HbsAg, positive vs. negative	1.27	0.67–2.40	0.460	1.29	0.62–2.69	0.488
HBV-DNA, >103 vs. ⩽103 IU/mL	1.08	0.63–1.87	0.776	1.94	1.13–3.34	0.017
AFP, ⩾400 ng/mL vs. <400 ng/mL	1.32	0.85–2.05	0.221	1.64	1.06–2.53	0.026
Preoperative ALT, IU/L	1.00	0.99–1.01	0.914	1.00	0.99–1.01	0.926
Preoperative AST, IU/L	1.00	1.00–1.01	0.341	1.00	1.00–1.01	0.248
ALBI Grade, Grade 2 vs. Grade 1	1.80	1.15–2.82	0.011	1.07	0.67–1.71	0.774
FIB-4 score						
Grade 2 vs. Grade 1	1.59	0.85–2.99	0.150	1.07	0.54–2.10	0.849
Grade 3 vs. Grade 1	1.45	0.76–2.78	0.258	1.40	0.73–2.65	0.309
BCLC Classification						
BCLC-B vs. BCLC-A	1.25	0.70–2.24	0.450	1.98	1.15–3.40	0.014
BCLC-C vs. BCLC-A	3.99	1.58–10.07	0.003	3.50	1.59–7.70	0.002
AJCC-TNM Stage						
Stage II vs. stage I	1.75	1.05–2.93	0.032	2.00	1.17–3.42	0.012
Stage III vs. stage I	1.21	0.70–2.09	0.491	2.92	1.73–4.93	<0.001
Tumor differentiation, poor vs. good	1.36	0.88–2.11	0.168	1.28	0.83–1.98	0.263
MVI, yes vs. no	2.07	1.33–3.22	0.001	1.98	1.27–3.08	0.003
Stromal type, type B vs. type A	2.94	1.79–4.85	<0.001	1.77	1.11–2.80	0.016

HBV, hepatitis B virus; AFP, alpha fetoprotein; ALT, alanine aminotransferase; AST, aspartate aminotransferase; ALBI, albumin-bilirubin; FIB-4, Fibrosis 4 Score; BCLC, Barcelona Clinic Liver Cancer; AJCC, American Joint Committee on Cancer; MVI, microvascular invasion.

Supplementary Table 5. Univariate analysis in the testing cohort.

Variables	Overall survival			Disease-free survival		
	HR	95%CI	P	HR	95%CI	P
Age, years	1.00	0.98–1.02	0.986	0.99	0.97–1.01	0.190
Gender, female vs. male	0.82	0.43–1.53	0.527	0.49	0.25–0.95	0.035
HbsAg, positive vs. negative	1.55	0.72–3.36	0.266	1.72	0.83–3.57	0.146
HBV-DNA, $>10^3$ vs. $\leqslant 10^3$ IU/mL	2.30	1.28–4.13	0.006	0.95	0.56–1.61	0.861
AFP, ⩾400 ng/mL vs. <400 ng/mL	1.05	0.68–1.60	0.840	1.79	1.16–2.79	0.009
Preoperative ALT, IU/L	1.00	1.00–1.01	0.129	1.00	1.00–1.01	0.397
Preoperative AST, IU/L	1.01	1.00-1.01	0.032	1.00	1.00–1.01	0.350
ALBI Grade, Grade 2 vs. Grade 1	1.46	0.94–2.26	0.088	1.44	0.90–2.30	0.126
FIB-4 score						
Grade 2 vs. Grade 1	1.30	0.69–2.44	0.414	1.28	0.71–2.32	0.412
Grade 3 vs. Grade 1	1.39	0.75–2.57	0.291	1.16	0.63–2.13	0.640
BCLC Classification						
BCLC-B vs. BCLC-A	2.37	1.42–3.94	<0.001	1.57	0.90–2.72	0.113
BCLC-C vs. BCLC-A	2.44	1.05–5.67	0.038	2.60	1.04–6.52	0.042
AJCC-TNM Stage						
Stage II vs. stage I	2.45	1.46–4.12	<0.001	2.13	1.28–3.53	0.003
Stage III vs. stage I	2.82	1.69–4.73	<0.001	1.30	0.74–2.28	0.365
Tumor differentiation, poor vs. good	1.13	0.74–1.72	0.575	1.01	0.64–1.58	0.969
MVI, yes vs. no	2.09	1.37–3.20	<0.001	2.22	1.43–3.46	<0.001
Stromal type, type B vs. type A	1.58	1.01–2.46	0.044	2.47	1.51–4.05	<0.001

HBV, hepatitis B virus; AFP, alpha fetoprotein; ALT, alanine aminotransferase; AST, aspartate aminotransferase; ALBI, albumin-bilirubin; FIB-4, Fibrosis 4 Score; BCLC, Barcelona Clinic Liver Cancer; AJCC, American Joint Committee on Cancer; MVI, microvascular invasion.

Supplementary Table 6. Univariate analysis in the validation cohort.

Variables	Overall survival			Disease-free survival		
	HR	95%CI	P	HR	95%CI	P
Age, years	1.00	0.96–1.04	0.946	0.99	0.96–1.02	0.446
Gender, female vs. male	2.30	0.76–6.94	0.140	1.36	0.51–3.67	0.539
HbsAg, positive vs. negative	1.82	0.42–7.85	0.424	0.91	0.37–2.25	0.834
HBV-DNA, >103/≤103 IU/mL						
AFP, ≥400 ng/mL vs. < 400 ng/mL	2.05	0.85–4.97	0.111	2.03	0.94–4.41	0.072
Preoperative ALT, IU/L	1.00	0.99–1.01	0.715	0.99	0.98–1.00	0.121
Preoperative AST, IU/L	1.00	1.00–1.01	0.252	1.00	0.99–1.01	0.555
ALBI Grade, Grade 2 vs. Grade 1	1.48	0.53–4.12	0.453	1.20	0.51–2.82	0.677
FIB-4 score						
Grade 2 vs. Grade 1	4.20	0.54–32.55	0.170	0.69	0.27–1.75	0.431
Grade 3 vs. Grade 1	4.29	0.54–34.29	0.170	0.97	0.37–2.53	0.952
BCLC Classification						
BCLC-B vs. BCLC-A	1.77	0.62–5.07	0.289	0.93	0.40–2.15	0.869
BCLC-C vs. BCLC-A	2.92	0.98-8.70	0.054	1.79	0.72–4.47	0.209
AJCC-TNM Stage						
Stage II vs. stage I	1.77	0.47–6.58	0.397	1.50	0.59–3.80	0.395
Stage III vs. stage I	2.53	0.67–9.53	0.169	1.10	0.38–3.15	0.866
Tumor differentiation, poor vs. good	2.45	0.80–7.51	0.117	1.89	0.88–4.08	0.103
MVI, yes vs. no	2.29	0.88–5.98	0.090	2.90	1.41–5.96	0.004
Stromal type, type B vs. type A	3.44	1.42–8.38	0.006	2.84	1.37–5.89	0.005

HBV, hepatitis B virus; AFP, alpha fetoprotein; ALT, alanine aminotransferase; AST, aspartate aminotransferase; ALBI, albumin-bilirubin; FIB-4, Fibrosis 4 Score; BCLC, Barcelona Clinic Liver Cancer; AJCC, American Joint Committee on Cancer; MVI, microvascular invasion.

Supplementary Table 7. Multivariable analysis in the testing cohort.

Variables in the final model	Overall survival			Disease-free survival		
	HR	95%CI	P	HR	95%CI	P
AFP, ⩾400 ng/mL vs. < 400 ng/mL				1.41	0.89–2.22	0.142
ALBI Grade						
Grade 2 vs. Grade 1	3.14	1.29–7.64	0.012			
BCLC Classification						
BCLC-B vs. BCLC-A	1.57	0.91–2.73	0.107	1.65	0.95–2.87	0.076
BCLC-C vs. BCLC-A	2.63	1.08–6.42	0.034	3.05	1.33–6.99	0.009
MVI, Yes vs. no	1.72	1.09–2.71	0.021	1.54	0.95–2.50	0.081
Stromal type, type B vs. type A	2.27	1.39–3.69	0.001	1.81	1.14–2.90	0.013

AFP, alpha fetoprotein; ALBI, albumin-bilirubin; BCLC, Barcelona Clinic Liver Cancer; MVI, microvascular invasion. HR, hazard ratio; CI, confidence interval.

Supplementary Table 8. Multivariable analysis in the validation cohort.

Variables in the final model	Overall survival			Disease-free survival		
	HR	95%CI	P	HR	95%CI	P
AFP, ≥400 ng/mL vs. < 400 ng/mL				2.42	1.13–5.19	0.023
ALBI Grade						
Grade 2 vs. Grade 1	3.09	0.89–10.69	0.075			
BCLC Classification						
BCLC-B vs. BCLC-A	2.54	0.88–7.34	0.085	2.30	1.00–5.30	0.049
BCLC-C vs. BCLC-A	9.91	2.60–37.74	<0.001	8.59	1.97–37.55	0.004
MVI, Yes vs. no	1.48	0.91–2.38	0.112	2.57	1.12–5.91	0.026
Stromal type, type B vs. type A	3.80	1.37–10.55	0.011	2.59	1.21–5.58	0.015

AFP, alpha fetoprotein; ALBI, albumin-bilirubin; BCLC, Barcelona Clinic Liver Cancer; MVI, microvascular invasion. HR, hazard ratio; CI, confidence interval.
